# Supplementary material for: Protein Structural Information and Evolutionary Landscape by In Vitro Evolution
Source: Mol Biol Evol. 2019 Oct 31;37(4):1179–92. doi: 10.1093/molbev/msz256 (PMC7086169; doi:10.1093/molbev/msz256)
Supplement: msz256-Supplementary_Data [file msz256-supplementary_data.zip › msz256-Suppl_Data/suppl material dedca 28August2019.pdf]

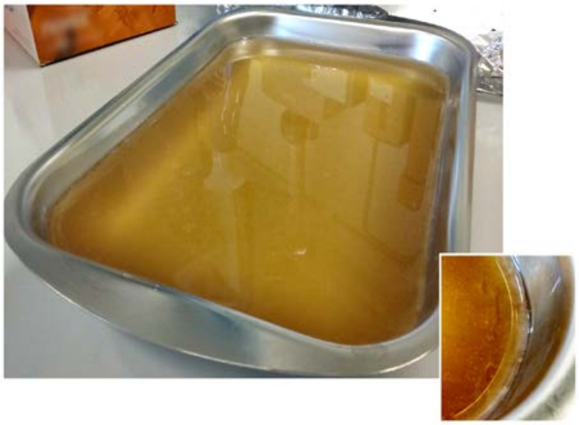

Supplementary figure S1 – Bacterial culture setup and colonial growth on Seaprep ultralow gelling soft agarose. Metallic tray containing 1L of bacterial culture grown in semisolid media. Small box on the right: high contrast image that reveal the colonial growth (the small white dots that can be seen in the growth medium).

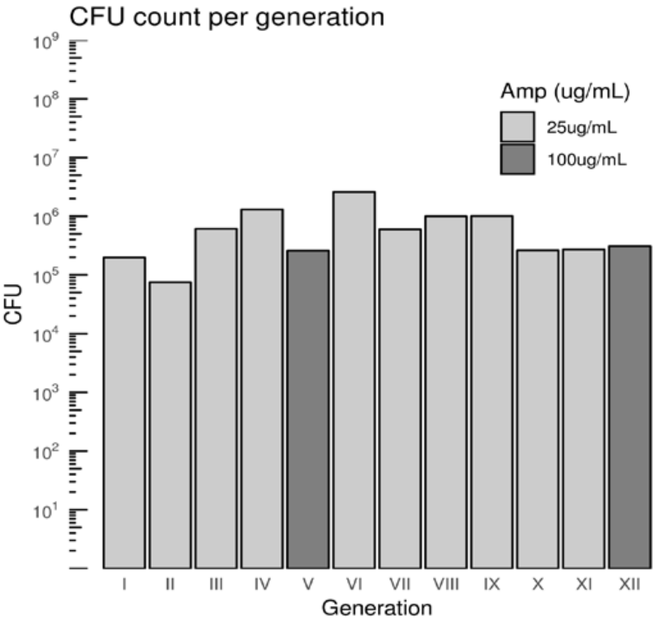

Supplementary figure S2 – Number of transformants obtained each generation. The number of transformants is obtained by collecting and plating on solid medium a small 1mL aliquot of the 1L bacterial culture before the gelification of the semisolid growth medium. The number of colonies present in the semisolid medium can be calculated from the CFUs observed in the aliquot sample. Due to technical problems it was not possible to obtain a precise colony count for the seventh (VII) generation and the value shown is only a rough estimation.

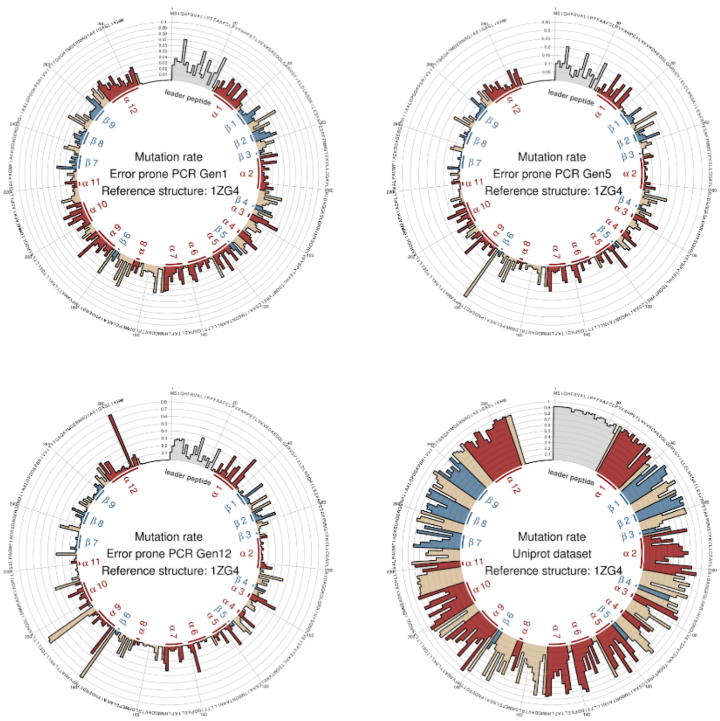

Supplementary figure S3 – Mutation rate per residue in the molecular evolution and in the uniprot dataset. The colors and annotations follow the secondary structure classification present in the PDB structure 1ZG4 (red: alpha helices, blue: beta strands, tan: coils). The leader peptide sequence (light ray) is missing in the structure.

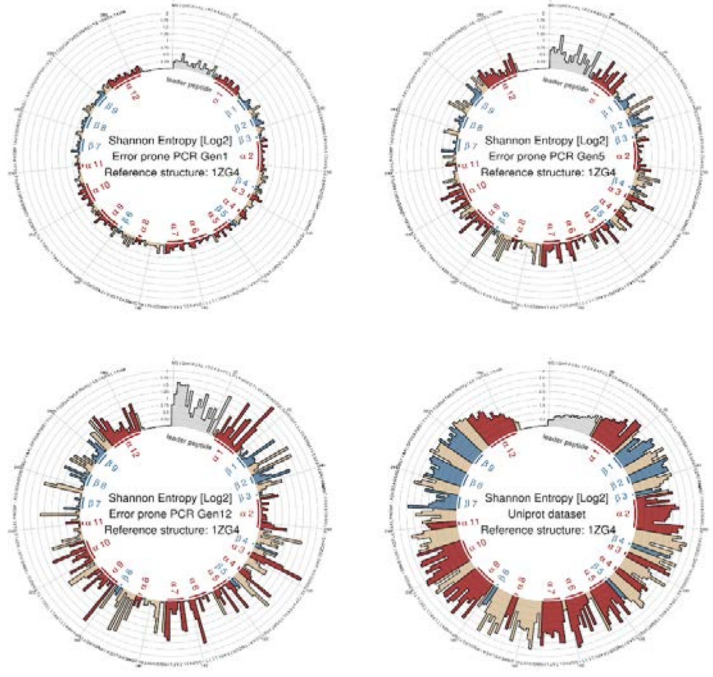

Supplementary figure S4 – Shannon entropy per residue in the molecular evolution and in the uniprot dataset. The colors and annotations follow the secondary structure classification present in the PDB structure 1ZG4 (red: alpha helices, blue: beta strands, tan: coils). The leader peptide sequence (light gray) is missing in the structure.

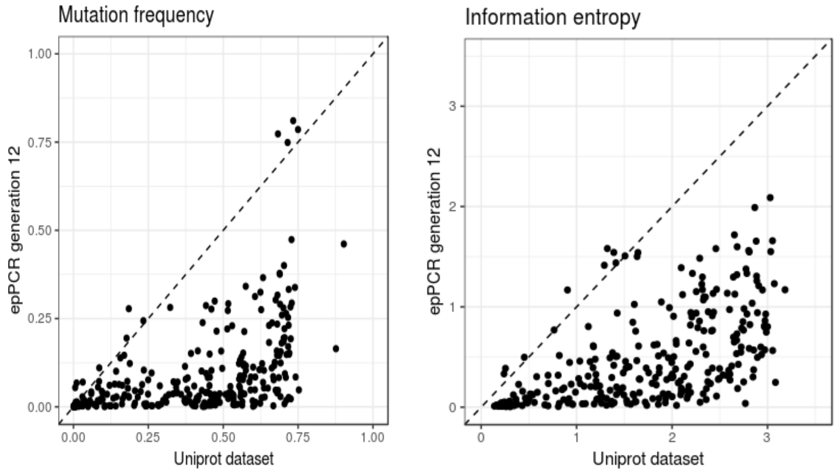

Supplementary figure S5 – Comparison of the Shannon entropies and mutation rates observed between positions of the uniprot dataset and the same position of the twelfth generation of molecular evolution. Correlation of the Shannon entropies (right) and mutation rates (left) observed between positions of the uniprot dataset and the same position of the twelfth generation of molecular evolution.

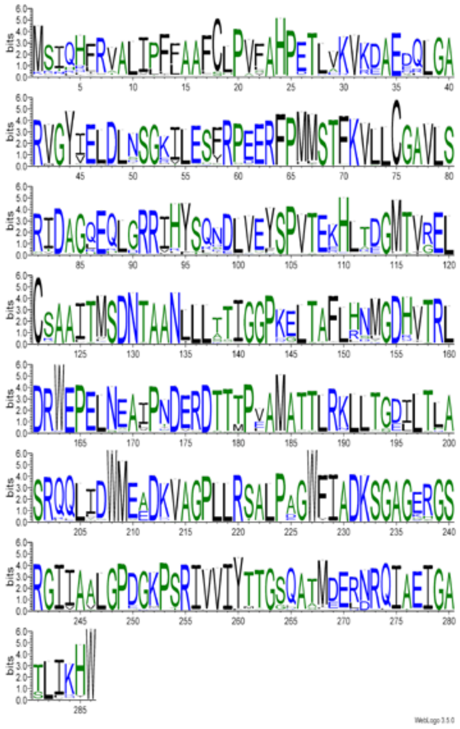

Supplementary figure S6 – Logo representation of the conservation in the amino acid sequence after 12 generations of molecular evolution.

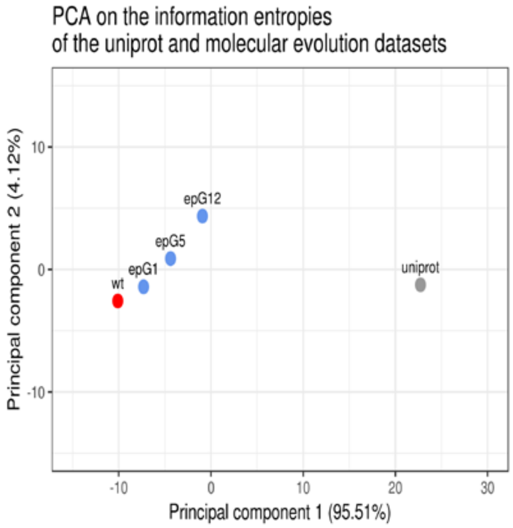

Supplementary figure S7 – Principal component analysis applied to the uniprot and molecular evolution dataset. Each point represent a dataset. Shannon information entropy was calculated for each position of each dataset and then subjected to PCA. Euclidean distance was used as distance metric. Gray represent the uniprot dataset, cyan the molecular evolution libraries (ep: error prone PCR). Numbers in the labels above the data points indicate the molecular evolution generation. The original pUC19 TEM-1 beta lactamase (wt: "wild type", in red) was added as additional datapoint before the analysis as a zero vector. The percentage of variance (POV) of the component is shown in brackets on the axis label.

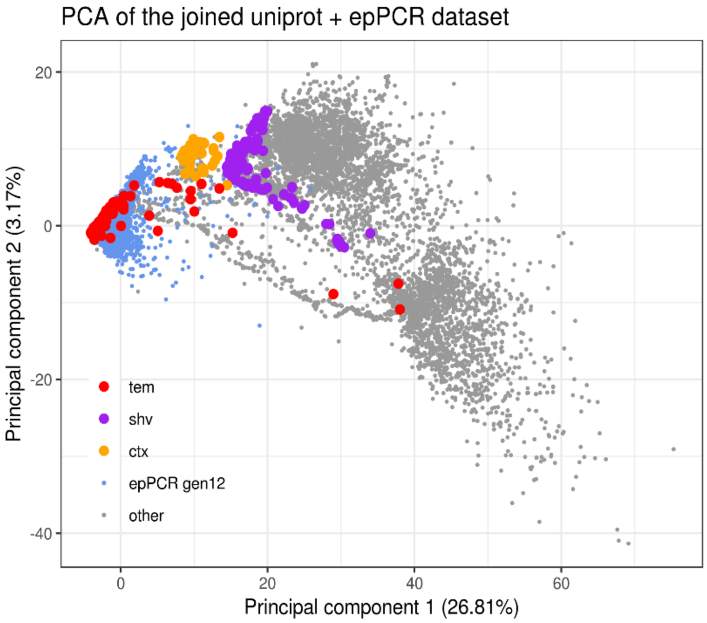

Supplementary figure S8 – Principal component analysis applied to the joined uniprot / error prone PCR 12th generation library dataset. Each point represent a sequence of the joined dataset. Each amino acid in the sequence was encoded as the frequency of that amino acid in that position in the entire joined dataset. The frequency value of each position was ranked and then subjected to PCA. Euclidean distance between ranks was used as distance metric. Gray and cyan represent the original dataset (gray uniprot, cyan epPCR library). Overlayed on top, the uniprot sequences' membership to one of the three main families of type A beta lactamases retrieved from the corresponding uniprot annotation are displayed in bright colours. The original pUC19 beta lactamase before molecular evolution is classified as a TEM beta lactamase (red). The percentage of variance (POV) of the component is shown in brackets on the axis label

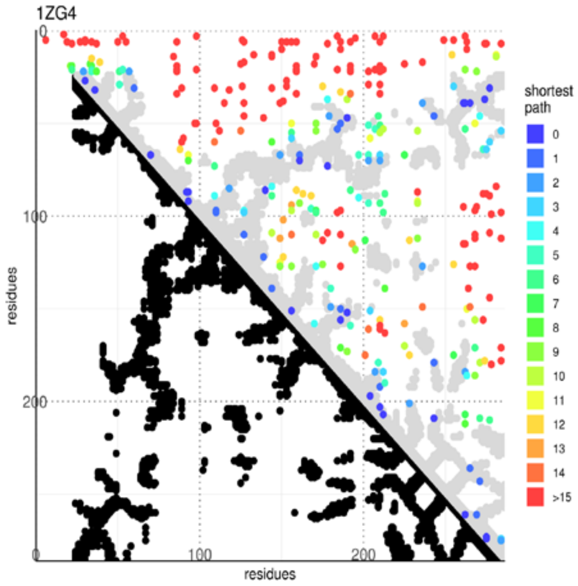

Supplementary figure S9 – DCA plot of the first generation library. DCA plot showing the top L (L = 286, the length of the protein amino acid chain) contact predictions by DCA obtained from the first generation of molecular evolution. The graph is an LxL grid where each axis represents the amino acid positions of the lactamase chain, from the N- to C-terminals. Each point represents the pair of residues described by its coordinates. The graph is separated in two halves. In the lower half black dots represent pairs of residues that have at least a pair of their respective non-hydrogen atoms less than 8.5Å apart in the reference crystallographic structure (PDB id: 1ZG4). These positions are considered residues in contact with each other. In the upper half the top L DCA predictions from the molecular evolution dataset are plotted above the gray mirrored silhouette of the crystallographic contacts. Pairs where the respective residues are less than 5 positions apart in the lactamase alignment are excluded from this ranking to promote visualization of long range interactions. In the graph the color indicates the shortest path (as the lowest L1 norm in the graph grid space) connecting the point to a contact pair position (a pair of residues that have non-hydrogen atoms less than 8.5Å apart in the reference structure).

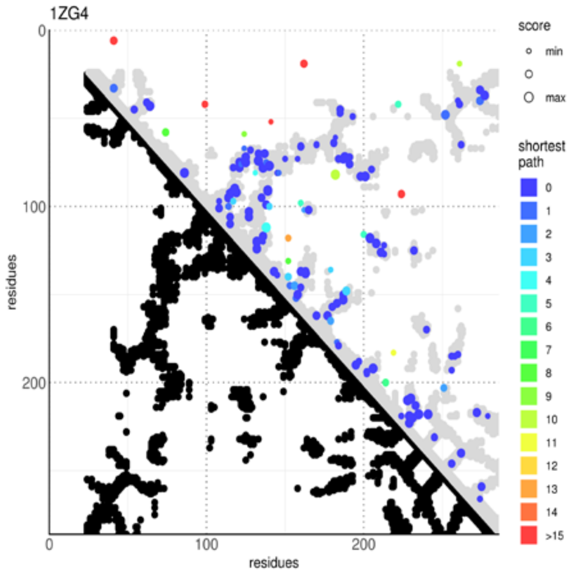

Supplementary figure S10 – Partial correlation of the uniprot dataset. Plot of the top L/2 partial correlations of residue positions on DCA score obtained from the uniprot dataset.

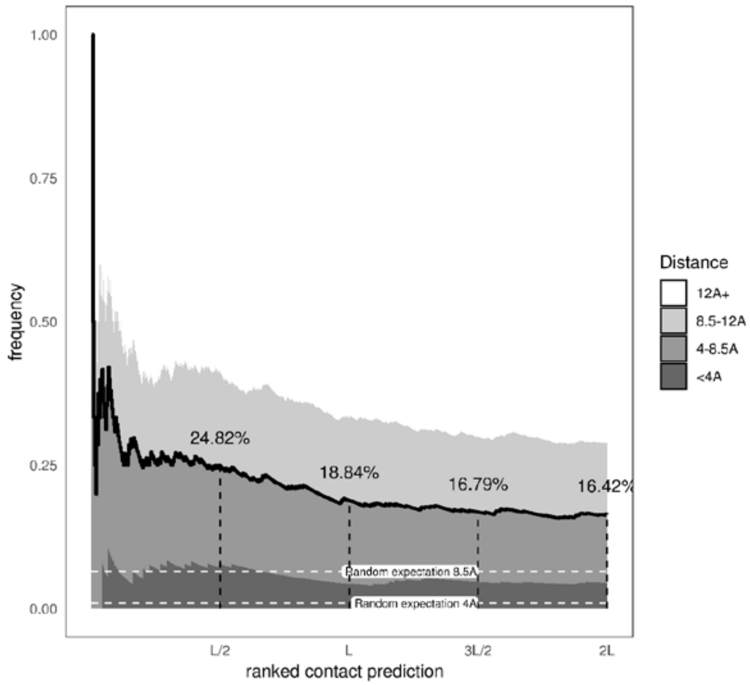

Supplementary figure S11 – Accuracy of the partial correlation prediction using different distance thresholds to define the residues in contact. Every possible combination of residues pair of the TEM-1 lactamase is sorted in a list following a rank determined by value of the partial correlations generated in the 12th generation of molecular evolution (bigger first). This list is converted in a binary format where the value depends if the residue pair is in contact in the reference structure 1ZG4. Residue pairs are defined in contact if they have atoms less than 4, 8.5 or 12Å apart in the reference structure. Taking an increasing number of elements from the top of this list, the graph shows the percentage of these that are TRUE contacts, i.e. the accuracy of the contact prediction for the sample size considered. Dotted white lines represent the frequency of position pairs expected to be under 4 and 8.5Å apart in a random sampling (random expectation). L = 286.

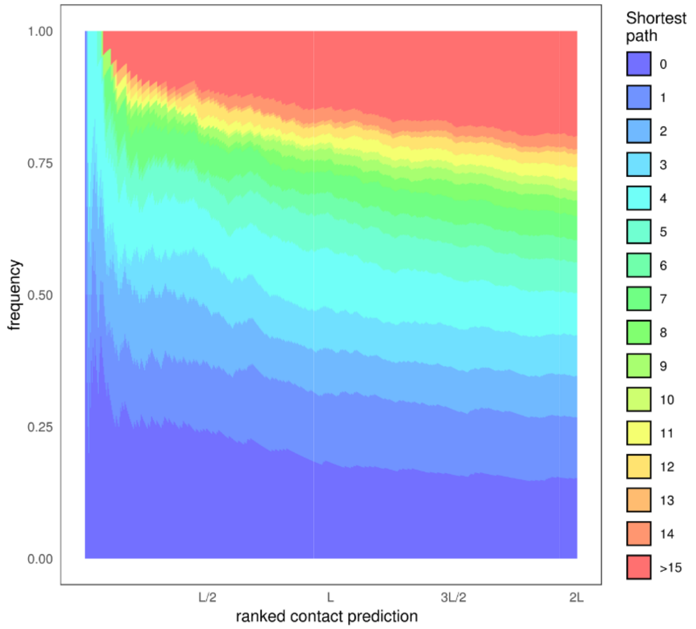

Supplementary figure S12 – Accuracy of the partial correlation prediction considering the shortest path to reach a contact. The residue pairs of the beta lactamase where ranked and sorted in a list as described in Supplementary figure S11. This list is converted in a binary format where the TRUE is assigned if the distance (shortest path) of the residue pair to the nearest pair considered in contact in the reference structure 1ZG4 is at or below a certain number (0-15). Residue pairs are defined in contact if they have atoms less than 8.5Å apart in the reference structure. Taking an increasing number of elements from the top of this list, the graph shows the percentage of these that are TRUE, i.e. the accuracy of the contact prediction for the sample size considered in different shortest path groups. L = 286.

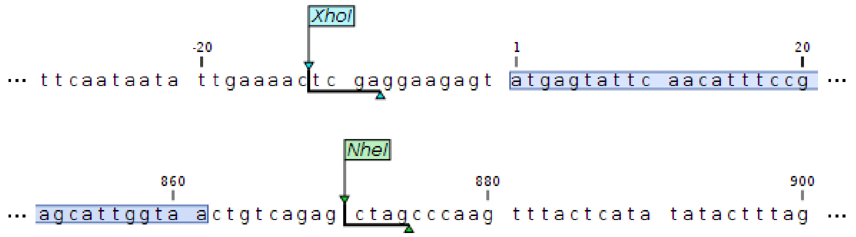

Supplementary figure S13 – pUC19a beta lactamase starting and terminal sites. The DNA sequence corresponding to the lactamase open reading frame is highlighted in blue. Restriction sites for the XhoI and NheI enzymes used during molecular evolution are shown in cyan and green.

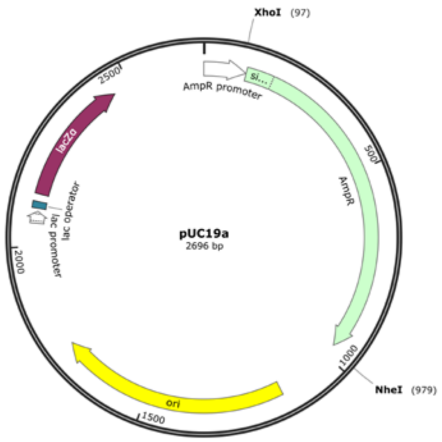

Supplementary figure S14 – Plasmid pUC19a with features and relevant restriction enzymes.
